# Prox1 Facilitates Transfected CHO Cell Proliferation through Activation of the AKT Signaling Pathway

**Published:** 2010-03

**Authors:** Shang-Zhi Xu

**Affiliations:** *Department of Pulmonary & Critical Care Medicine, College of Medicine, Mayo Clinic, 200 First Street SW, Rochester, MN, USA*

**Keywords:** AKT, cell proliferation, CHO, Prox1 (gene), Prox1 (protein), PI3K

## Abstract

The homeobox transcription factor Prox1 plays an important role in the development of many embryonic organs. Previous studies indicated that Prox1 facilitates hepatic progenitor-cells proliferation. However, the underlying mechanism of Prox1 in tumor genesis, formation, and progression are poorly understood and need to be exploited. Herein, Chinese Hamster Ovary (CHO) cells were transfected and over-expressed human recombinant Prox1 gene, and developed several stable cell lines of Prox1-CHO after screening. The results indicated that over expression of Prox1 increased CHO cell proliferation in comparison to GFP-CHO and parental CHO cells, and Prox1 increased AKT phosphorylation and up-regulated PI3 Kinase expression. An AKT specific inhibitor-AKTi—X (5 μM) and a PI3 K inhibitor—LY294002 (5 μM) were able to reverse AKT phosphorylation and PI3 K expression induced by Prox1, respectively. Furthermore, AKTi-X but LY-294002 decreased Prox1-CHO cell proliferations at 48 and 72 h. Our results suggest that over expression of Prox1 facilitates CHO cell proliferation via activation of the AKT signaling pathway. This finding provides new insights into the mechanism of Prox1 mediated tumor growth and metastasis where Prox1 is rich.

## INTRODUCTION

The Prospero-related homeobox1 (Prox1) is a member of the divergent Prospero (Pros) family of transcription factor, for which is featured with homeodomain (HD) flanked with Prospero domain (PD). The combined structural unit of HD and PD is called Homeo-Prospero domain (HPD) (1–2). The gene of Prox1 has been widely found in human embryonic developmental organs such as lens, brain, liver, etc, but postnatal expressions of Prox1 are limited in liver and heart. Prox1 is composed of 5 exons, mapped in chromosome 1q32.2–q32.3 and its full length of (2.3 kb) cDNA encoded a full length protein of 736 amino acids (83 kDa). Prox1 shares 89–94% sequence identities across different vertebrates, e.g., Xenopus, zebrafish, chickens, human, but displays great variances of mRNA spliced forms across different species, different tissues, and during different developmental ages at the same tissue (3–4).

It is well known that Prox1 is a key control gene in re-programming genotype of venous endothelial cells by up-regulating genes of lymphatic endothelial cells, whereas down-regulating specific genes of vascular endothelial cells (5). If Prox1 is transfected in blood vascular endothelial cells (BVEC), the phenotype of BVEC is gradually changed; the lymphatic markers such as vascular endothelial growth factor receptor (VEGFR-3) in BVEC are dominant while the blood vascular markers such as collagen IV and lamnin are weak (6). In Prox1 null murine embryos, buddings from veins that gives rise to the lymphatic vasculature are showed lack of any lymphatic markers such as VEGFR-3, lymphatic endothelial hyaluronan receptor (LYVE-1) and secondary lymphoid chemokine (SLC) (7).

Recently Prox1 has received great attention because it demonstrates that it plays a more important role in progression of various carcinomas than before. Previous cases screening data from a cohort study revealed that Prox1 was either silenced by hypermethylation in hematologic malignances and breast cancers, or deleted in bilary and hepatocellular carcinomas, or point mutated from A to G base in esophageal cancer. (8–13). However, recent studies suggest that Prox1 is more likely to facilitate tumor formation and accelerate tumor progression. Besides participating in the development of lymphatic vasculature and retina, lens and brain organ-genesis, Prox1 increases hepatic stem/progenitor cells proliferation and facilitates these cells migration. (4, 7, 8, 14–19). New data (20) evidenced that the appearance of Prox1 is the hallmark of the transition from benign colon adenoma to malign carcinoma, and over expression of Prox1 promotes intestinal tumor progression. In our Lab, we recently found Prox1 is highly expressed in small cell lung cancer (SCLC) patients and several derived cell lines. If Prox1 was knocked down with shRNA lentivirus, proliferative rates of SCLC were dramatically decreased (data not shown here).

The current study aims at investigating the effect of over expressed Prox1 on cell proliferation, and unveiling associated potential signaling pathways. Our results indicated that over expression of Prox1 increases AKT phosphorylation and PI3 kinase expression, thereby increasing transfected CHO cells proliferation via activation of AKT signaling pathway; thus, it opens a new avenue to intervene in tumor progression and metastasis in the future.

## MATERIALS AND METHODS

### Materials

Prox1 (full length: 2.3Kb) vector with EGFP plasmid (Cat#EX-F0925-M03), empty vector with EGFP plasmid (Cat#EX-EGFP-M03) were purchased from Gene-Copoeia, Inc. (Germantown, MD 20874). Plasmid DNA was expanded and purified by using S.N.A.P MidiPrep Kit (Cat#K1910-01, Invitrogen). Library efficiency DH5α competent cells used for transformation, Lipofectamine 2000 (Cat#11668-049) used for transfection, Prolong gold antifade reagent with DAPI used for nuclear staining (Cat#P36931) were all purchased from Invitrogen. Prox1 antibody (Cat#51043-1-AP) was purchased from Proteintech Group, Inc. (Chicago, IL 60612). AKT antibody (Cat#9272), phospho-AKT (Ser473) antibody (Cat#4058), PI3 Kinase (p85) (Cat#4292) were purchased from Cell Signaling Technology. Control β-actin antibody was purchase from Sigma. Pan Proteinase inhibitor cocktail was purchased from Roche Applied Science (Cat#11836153001). AKT inhibitor X (10-(4′-(N-diethylamino)buyl)-2-chlorophenoxazine, HCl, AKTi-X), PI3 Kinase inhibitor (LY294002, 2-(4-morpholinyl)-8-phenyl-4H-1-benzopyran-4-one) were purchased from Cayman Chemical. Cell proliferation Biotrak ELISA kit (Cat#RPN250), and ECL advance western blotting detection kit (Cat#RPN2135) were purchased from GE Healthcare. CHO cell line was purchased from ATCC.

### Plasmid DNA Preparation

Prox1-EGFP plasmid and EGFP control plasmid were transformed into DH5α competent cells. The right colonies were selected and confirmed by DNA sequencing. Plasmid DNA was expanded and prepared by using S.N.A.P. MidiPrep kit, and the DNA final concentration and purity were determined by a Nano drop Spectrophotometer (ND-100).

### Plasmid DNA Transfection

To create Prox1-CHO and GFP-CHO cell lines, Plasmid DNAs of Prox1-EGFP and empty vector-EGFP were transfected by using Lipofectamine 2000 in accordance with the manufacture’s instruction. Briefly, total 2 × 10^5^ cells/ml/well with DMEM medium (containing 10% heat inactivated fetal bovine serum (FBS), 100 U/ml penicillin, and 100 μg/ml streptomycin) was freshly prepared and seeded in a 6-well plate. After growing overnight, cell density reached about 75% confluence. The ratio of 4 μg DNA and 8 μl Lipofectamine 2000 were set up in 50 μl Opti-Mem Medium separately. Before transfection, the cells were washed once with DMEM (pH7.2) without serum and antibiotics, and the final volume was 1 ml of that medium. One hundred micro liter mixture of DNA and Lipofectamine 2000 were added into each well in the 6-well plate After 6 h incubation, the medium was exchanged with 2 ml of 10%FCS DMEM without antibiotics. The efficiency of transfection was determined by GFP expression via fluorescent microscopy after 24h incubation. In general, about 30–50% cells efficiency of transfection were obtained.

### Cell Line Development

After 48 h transfection, adherent cells were harvested with 0.05% trypsin/EDTA. Prox1-CHO and GFP-CHO cells were selected by FACS and then individual green fluorescent cell was planted in each well of a 96-well plate. After a week, individual, well-separated green fluorescent cell colonies were picked up manually with a pipette tip and transferred to a 24-well plate; after another week, green fluorescent cell colonies were screened again and transferred to another 6-well plate. Thus, about 95–98% cells were expressed either Prox1-GFP or GFP only. The above screening process were repeated several times until the at least 98% purity of cells were obtained. Ten to twelve cell lines of Prox1-CHO and GFP-CHO were confirmed by the fluorescent microscopy and western blotting, and maintained for at least 3 generations before considered as stable cell lines. The highest GFP expressed of Prox1-CHO and GFP-CHO cell lines (Prox1-CHO-H1 and GFP-CHO-H1) were chosen for the application in the following experiments, and the rest cell lines were harvested and stored liquid nitrogen.

### Fluorescent Microscopy Observation

To acquire GFP images from living cells, Prox1-CHO, GFP-CHO, and parental CHO cells were seeded in 60 mm dishes, separately. Phase contrast and fluorescent images were simultaneously taken at 100 times magnification. In order to obtain high magnified and resolution images, the same three cell lines were seeded on 1% poly-D-lysine pre-coated coverslips in a 6-well plate. The cells were fixed with 4% paraformaldehyde at 37°C for 15 min, and then blocked with 3% bovine serum albumin (BSA) for 30 min, permeated with 0.2% Triton X-100 in PBS for 10 min at room temperature. The cover slips were completely dried and mounted on the slides with prolong gold antifade reagent with DAPI. The images were captured by a Zeiss 510 confocal microscope.

### Western Blotting

The standard procedure was employed from our previous study (21). Briefly, cells were lyzed with RIPA buffer (150 mM sodium chloride, 1% Triton X-100, 0.5% sodium deoxycholate, 0.1% sodium dodecyl sulphate (SDS), 50 mM Tris (pH8.0) with 2 mM PMSF and proteinase inhibitor cocktail. The suspension of cell lysates were sonicated, and centrifuged at 15,000 × g for 10 min at 4°C. The milky suspension was collected as measuring samples and the cell debris discarded. Protein concentration was determined using Lowry’s method (22). Equal amount of proteins was separated by 7.5% SDS-PAGE gel and transferred on to PVDF membrane. The membrane was probed with a polyclonal Prox1 antibody, and developed with chemiluminescent medium and exposed on X-ray film. The bands on films were scanned by using Un-scan-it software from Silk Scientific, Inc (Orem, Utah). Colorimetric density values of individual bands were determined by the same software, and subjected to statistical analysis by using One-way ANOVA program (SigmaStat 3.0). Each independent experiment was repeated three times.

### Cell Proliferation Assay

Cell proliferative rate was determined by using Cell proliferation Biotrak ELISA kit. The quantities of proliferated cells are determined by the amount of 5-bromo-2′-deoxyuridine (BrdU) incorporated into newly synthesized DNA. To determine the effect of over-expressed Prox1 on cell proliferation, equal number of cells at 1 × 10^5^ cells/well were seeded in a 24 well plate, and allowed to grow for 12h. In the paralleled experiment to acquire the effect of AKT inhibitor-AKTi-X and P13K inhibitor-LY294002 on cell proliferation for a longer time, the number of 1 × 10^4^ cells/well were seeded in a 24 well plate, and the culture was extended for 48 and 72h, separately. AKTi-X was dissolved in DDW. LY294002 was dissolved in pure ethanol. The final ethanol concentration in the medium was controlled below 0.1%, and the same amount of ethanol was used in the control cells. Fifty micromole of BrdU were added into it and incorporated for 2 h before harvesting cells. The monolayer of cells was undergone a sequential process of fixation, washing, and blocking, and then incubated with peroxidase-labeled anti-BrdU antibody at 37°C for 90 min. Eventually the color development was initiated with TMB (3,3′,5,5′-tetramethylbenzidine) substrate, and terminated with sulphuric acid. Aliquots were transferred to a 96-well plate for reading at 450 nm on a Versa Max Tunable Microplate Reader (Molecular Device Corp.).

## RESULTS

### Mapping the localization of Prox1 in transfected CHO cells

**Figure 1:**
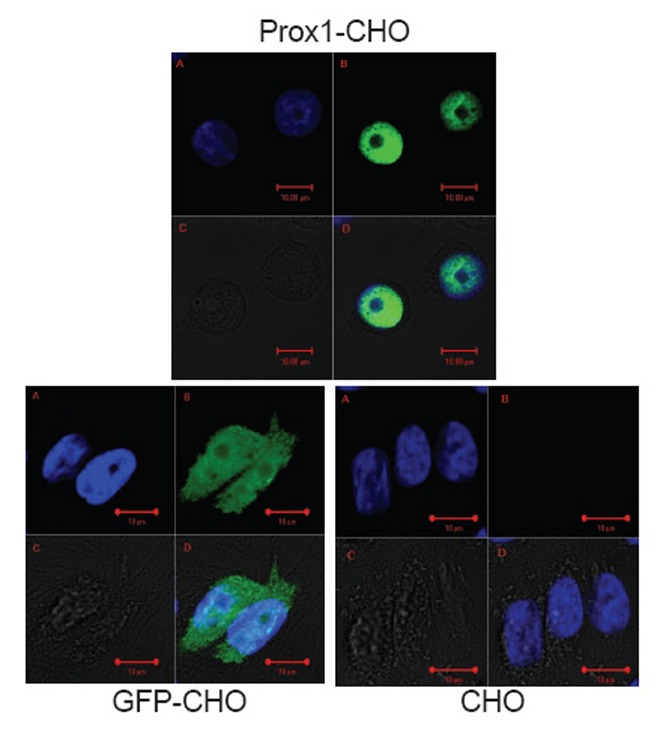
Human Prox1 abundantly expresses in the nuclei of CHO. Parental CHO, GFP-CHO and Prox1-CHO cells were examined with a Zeiss laser confocal Microscope. Prox1 highly concentrated in the nuclei, whereas control GFP distributed on the cell surface. Nucleuses were stained with DAPI.

**Figure 2:**
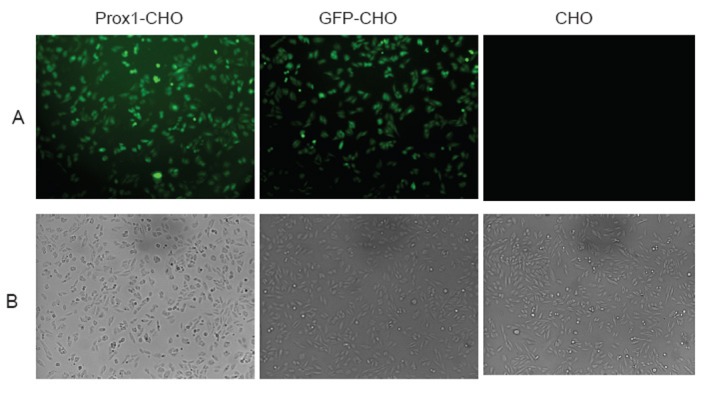
Fluorescent images of Prox1-CHO, GFP-CHO and parental CHO cell lines were examined under a fluorescent microscope. Both fluorescent and phase-contrast images were taken from living cells concurrently. (Magnify 100 X).

It has been reported that abundant Prox1 are found in the nucleus, but also exists in the cytoplasm (13). However, the prevailing location of Prox1 is diversified, which depend on different cell type of tumors, as well as differential stages of tumor (13, 22). As indicated in Fig. 1, Prox1 was observed highly concentrated in the nucleus of Prox1-CHO cells whereby the green color of GFP was shadowed by the blue color of DAPI staining of the nuclei. However, other distributions of Prox1 were also found in those images. For example, in some cells Prox1 were evenly distributed in the nucleus, whereas in other cells one part of Prox1 was aggregated in the nucleus and other part of Prox1 were scattered in the cytoplasm (images not shown). In living cells, Prox1 was found continuously danced with cells (movie not shown). In GFP-CHO cells, GFP was only showing on the cell surface of the cytoplasm; while in parental CHO cells, there was no green fluorescence at all. Subsequently, in low magnification images (Fig. 2), Prox1-CHO and GFP-CHO cells were shined with GFP fluorescence, while parental CHO cells were negative.

**Figure 3:**
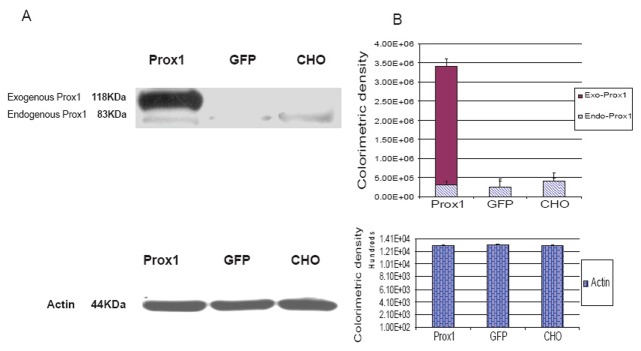
Over-expressed Prox1 in CHO cell lines was determined by western blotting. (A) Endogenous Prox1 was expressed at 83 KDa, whereas exogenous Prox1 (Prox1 83 KDa plus GFP 35 KDa) was shown at 118 KDa ; (B) Colorimetric densities of individual bands were expressed as mean ± SD and subjected to One-way ANOVA program and followed by Dunnett's test (SigmaStat 3.0). Recombinant Prox1 was only expressed in Prox1 transfected cell line. Endogenous Prox1 was determined between Prox1-CHO, GFP-CHO, and parental CHO cells. Actin protein was used as the control.

Meanwhile, Prox1 protein was analyzed with western blotting as shown in Fig. 3. Endogenous Prox1 was traced across three analyzed cell lines but no difference was found by the means of colorimetric densities, exogenous Prox1 was found only in Prox1-CHO cells but others. Actin protein was used as the control and the amount measurements of colorimetric densities on the bands were almost equal and did not show any statistical differences.

### Prox1 facilitated CHO cells proliferation

As mentioned before, Prox1 plays a pivotal role in the development of many embryonic organs and tissues. Over expression of Prox1 induced migration and proliferation of fetal hepatic stem/progenitor cells *in vitro*. Prox1 null mice died around embryonic days 14.5 and the liver was hypoplastic down to 70% of normal developmental size after necropsies (4, 14, 23). To determine whether Prox1 affected CHO cell proliferation, three cell lines of proliferation rate were assessed by BrdU incorporation. The amount of incorporated BrdU into newly synthesized DNA represents the number of divided cells, which is reflected on an absorbance at OD450nm when BrdU is probed by antibody and reacted with the substrate for color development. All three cell lines of parental CHO, GFP-CHO and Prox1-CHO were controlled to grow for 12 h. The results showed that Prox1-CHO cells grew much faster than GFP-CHO and parental CHO cells. Statistical differences were significant (P<0.01) when Prox1-CHO cells compared to GFP-CHO and parental CHO cells. Nevertheless, there was no statistical difference between GFP-CHO and parental CHO cells (Fig. 8A).

### Prox1 increased AKT phosphorylation and PI3 K expressions in transfected CHO cells

**Figure 4:**
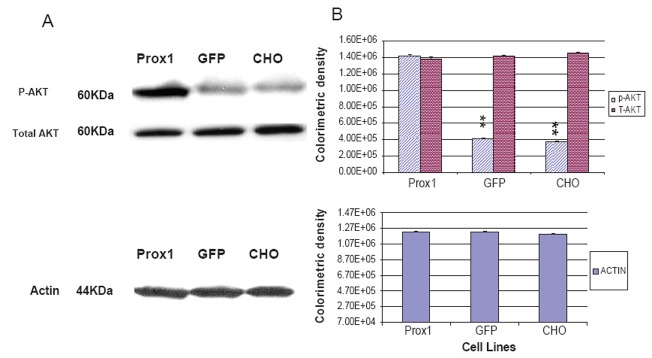
Prox1 increases phosphorylated-AKT in Prox1-CHO cells. (A) Total AKT and phosphor-AKT were determined by western blotting between Prox1-CHO, GFP-CHO and parental CHO cell lines; (B) Colorimetric densities of individual bands were expressed as mean ± SD and subjected to One-way ANOVA program and followed by Dunnett's test (SigmaStat 3.0). The statistical significance was indicated as **P<0.01 when GFP-CHO and parental CHO compared to Prox1-CHO. Actin protein was used as the control.

**Figure 6:**
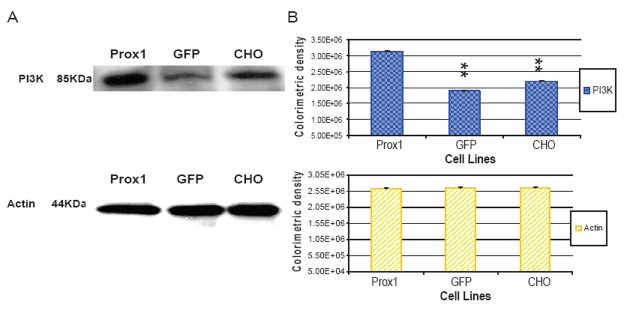
Prox1 induces over expression of PI3K in Prox1-CHO cells. (A) PI3K was determined by western blotting between Prox1-CHO, GFP-CHO and parental CHO cells; (B) Colorimetric densities of individual bands were expressed as mean ± SD and subjected to One-way ANOVA program and followed by Dunnett;s test (SigmaStat 3.0). The statistical significance was indicated as **P<0.01 when GFP-CHO and Parental CHO compared to Prox1-CHO. Actin protein was used as the control.

A recent study implied that both PI3 K- AKT and Prox1 are involved in regulation of lymphatic marker –VEGFR-3 and podoplanin expressions, and the gp130 receptor plays a central role in cross-talking with these two signal pathways (24). However, the dispute on a sequential order of signaling cascades remains to clarify. In this study, phosphorylated AKT (p-AKT) was greatly increased in Prox1 transfected CHO cells against GFP-CHO and parental CHO cells, whereas no significant difference between GFP-CHO and parental CHO cells (Fig. 4). However, total amount of AKT was consistent. PI3K, an upstream signaling molecule of AKT, was also found greatly increased in Prox1-CHO cells. Colorimetric density of PI3K in Prox1-CHO cells was much higher than in GFP-CHO and parental CHO cells (P<0.01). However, no statistical difference between GFP-CHO and parental CHO cells (Fig. 6). Other down stream signaling molecules, mTOR was slightly increased but no change of PTEN with over expression of Prox1 (data not shown).

### AKTi-X and LY294002 decreased Prox1 induced AKT and PI3K expressions

**Figure 5:**
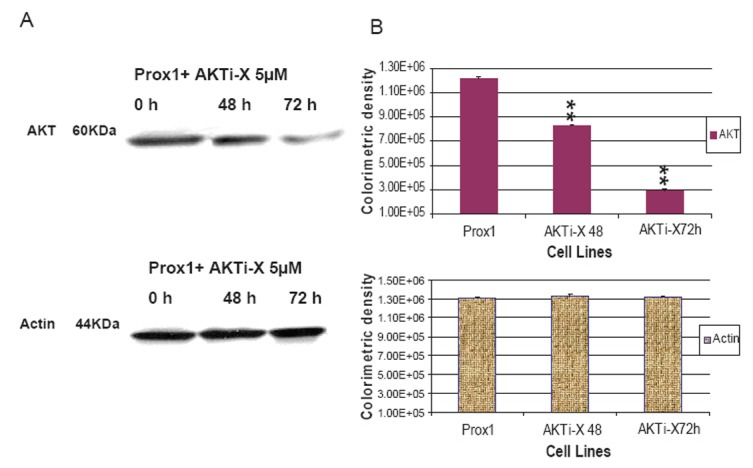
AKTi-X abrogated the increase of p-AKT in Prox1-CHO in a time dependent manner. (A) In the presence of AKTi-X (5 µM) for 48 h and 72 h, p-AKT in Prox1-CHO cells was determined by western blotting; (B) Colorimetric densities of individual bands were expressed as mean ± SD and subjected to One-way ANOVA program and followed by Dunnett's test (SigmaStat 3.0). The statistical significance was indicated as **P<0.01 when comparing AKTi-X treatment to non-treated Prox1-CHO. Actin protein was used as the control.

**Figure 7:**
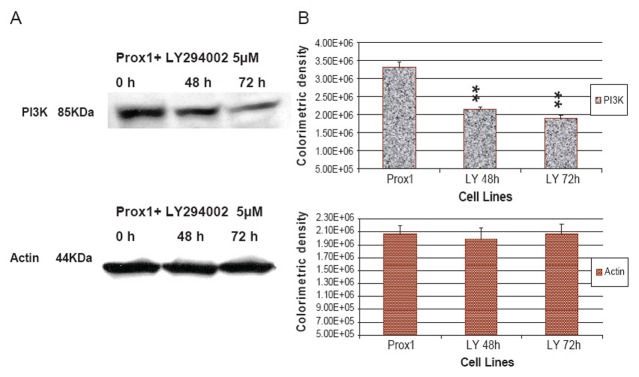
LY294002 abrogated the increase of PI3K in Prox1-CHO in a time dependent manner. (A) In the presence of LY294002 (5 µM) for 48 h and 72 h, PI3K in Prox1-CHO cells was determined by western blotting; (B) Colorimetric densities of individual bands were expressed as mean ± SD and subjected to One-way ANOVA program and followed by Dunnett's test (SigmaStat 3.0). The statistical significance was indicated as **P<0.01 when comparing LY294002 treatment to non- treated Prox1-CHO. Actin protein was used as the control.

AKTi-X specifically inhibits the AKT phosphorylation at the sites of Ser473 and Thr^308^(25). LY294002 not only inhibits the activity of PI3K, but also decreases its protein expression (26–27). Prox1 can up-regulate PI3K and increase p-AKT in transfected CHO cells, but it prompted us to ask whether AKTi-X and LY294002 were able to reverse such effects. As shown in Fig. 5, in the presence of AKTi-X (5 μM) for 48 h and 72 h. Phosphor-AKT (Ser^473^, p-AKT) was gradually decreased in a time-dependent manner. Likewise, LY294002 (5 μM) decreased PI3 K in the same manner (as seen in Fig. 7). Colorimetric densities at 48 and 72 h for p-AKT and PI3K were shown to have significant statistical differences as compared to untreated cells (P<0.01). Control protein-actin was consistent.

### AKTi-X but LY 294002 abrogated the increase of cell proliferation facilitated by Prox1

**Figure 8:**
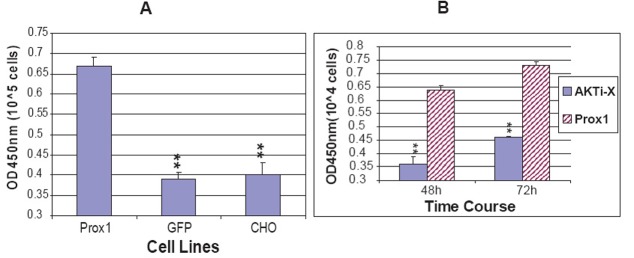
(A) Prox1 facilitates CHO cell proliferation. Three cell lines cell proliferation rates of Prox1-CHO, GFP-CHO and parental CHO were determined at 12 h by BrdU incorporation. The statistical significance was indicated as **P<0.01 when Prox1-CHO compared to GFP-CHO and parental CHO. (B) AKTi-X reverses the increase of cell proliferation rate induced by Prox1. Prox1-CHO cells were exposed to AKTi-X (5 μM) for 48 h and 72 h, cell proliferation rates were determined by BrdU incorporation. The statistical significance was indicated as **P<0.01 when comparing AKTi-X treatment to non-treated Prox1-CHO cells at 48 h and 72 h, respectively. The data were expressed as mean ± SD (n=4). Each independent experiment was duplicated.

Since AKTi-X and LY294002 decreased AKT and PI3 K in Prox1-CHO cells, it was speculated that AKTi-X and LY294002 reverse the increased rate of cell proliferation. As a result, AKTi-X (5 μM) indeed decreased cell proliferation rates at 48 h and 72 h, while LY294002 (5 μM) did not (Fig. 8B). If we increased LY294002 concentration, it killed Prox1-CHO cells soon (Data was not shown here). The reason might be associated the hydrophobic property of LY294002, and AKTi-X is hydrophilic and water soluble.

## DISCUSSION

In the present study, we have demonstrated that over expression of Prox1 greatly increases cells proliferation through activation of AKT signaling pathway. We have successfully over expressed human Prox1 with GFP tag in CHO cells. Transfections in other cell lines were transient and failed to establish stable cell lines. The role of Prox1 in transfected CHO cells was characterized compared with GFP-CHO (transfected with empty GFP vector) and parental CHO cells. It is indicated that over expression of Prox1 increases PI3K and enhances AKT phophorylation (p-AKT). Subsequently, a PI3K inhibitor-LY294002 and an AKT inhibitor-AKTi-X, abolishes such effects. Furthermore, the increased rate of cell proliferation is reversed by AKTi-X but LY294002. It suggests that Prox1 may facilitate cell proliferation and migration in tumor progression and metastasis.

In line with our results, Kamiya and his colleagues reported that over-expression of Prox1 by using retrovirus infection greatly increased fetal hepatocytic proliferation through suppressing the expression of p16^ink4a^, the cdk inhibitor (14). Many studies demonstrated that Prox1 is the master gene in determining the fate of embryonic endothelial cells and reprogramming them to differentiate towards lymphatic vasculature other than blood vasculature, as evidenced by up-regulating the expression of lymphatic endothelial cell (LEC) markers and down-regulating blood vascular endothelial cell (BEC) markers (6–7, 18–19, 28). As a matter of fact, not only lymphatic endothelial cells, but also postnatal and adult LEC are required to maintain lymphatic phenotype identity by certain amount of Prox1 activity. Loss or down regulated Prox1 in LEC would lead to LEC markers disappeared and BEC markers re-appeared (29). In addition, Prox1 activity is both necessary for progenitor-cell proliferation and determining cell fate in the vertebrate retina (30). In regard to embryonic development of liver and brain, previously the profile of Prox1 expression over different time and spatial patterns was obscure, but now it is loomed and becomes clear (4, 31–32).

The focus of Prox1 in physiological condition has been shifted to tumor genesis. For example, the lymphatic vessels pipeline tumor metastasis. Notably, Prox1 is emerged as a good target to treat tumor metastasis fundamentally. Prox1 alone is not able to trigger tumor genesis, but it is able to drive tumor progression via disruption of cell polarity and adhesion (20).

The advance of Prox1 underlying mechanism is struck. Lee and his co-workers (17) found that COUP-TFII functions as a co-regulator of Prox1 to control several lineage-specific genes including VEGFR-3, fibroblast growth factor receptor (FGFR)-3 and neuropilin-1 and is required along with Prox1 to maintain LEC-phenotype. Besides regulating other genes, Prox1 itself could be modified by a small ubiquitin-related modifier (SUMO)-1 to control its activity (33). Dysfunction of Prox1 disorders regulatory genes in controlling cell cycle (34). Functional mutations of Prox1 result in the transcriptional mis-regulation of many genes (32). Thus, it raises the assumption that Prox1 may cooperate with other transcription factors to regulate cell proliferation and function.

However, which domain of Prox1 exerts interactions with other transcription factors or DNA sequences? Indeed, except for HD and PD domains, Prox1 also has 5 repeats of poly-Gln and 5 sites of phosphor-serine and 1 phospho-threonine (http://ca.expasy.org/uniprot/Q92786). PD is deemed to bind DNA sequence (preferentially 5′-AAGACG-3′), and the underlined two bases are considered as the key consensus sequence for Prox1 binding. HD holds the defined nuclear exports signal (NES) that is masked by the flanking PD. If removing the blockade of PD, exposed NES would convey Prox1 from the nuclei to the cytoplasm, where Prox1 are highly phosphorylated and stay in an open status. If NES shielded by PD that is induced by a physiological signal, Prox1 would transform back into a close status and is able to enter the nuclei (1–2). For instance, partitioning and translocation of Prox1 to the GMC nucleus results in transcriptional termination of multiple cell-cycle regulatory genes, which effectively limits the capacity of a cell to undergo additional round of mitotic division, and then enter into terminal differentiation (34).

Prox1 facilitating cell proliferation makes great contributions to cancer progression. Petrova and her colleagues (20) recently showed that Prox1 promotes dysplasia in colonic adenomas and colorectal cancer (CRC) progression; loss of Prox1 does not prevent tumor initiation but instead impairs tumor progression. Their results suggested that Prox1 acts as an essential downstream effector of TCF/β-catenin signaling in CRC. Prox1 contributes to tumor progression by disrupting tissue architecture, cell polarity, and adhesion, which in the context of on-cogenic Wnt signaling leads to spatially unrestricted cell proliferation. In our present study, we present another signaling pathway (AKT) that conducts cell proliferation activated by over expression of Prox1. Recently, Morris and his co-workers (24) showed that blood endothelial cells (BEC) infected with Kaposi’s sarcoma-associated herpesvirus (KSHV) induce LEC differentiation. When they explored the underlying mechanism, they found several signaling pathways being activated. Activation of PI3 K/AKT cell signaling pathway is necessary for KSHV-induced lymphatic reprogramming of BEC. According to their results, the PI3 K inhibitor-LY294002 abolished a 3.5-fold higher mRNA of Prox1 induced by KSHV. However, they did not see any changes of p-AKT after insulted by Prox1 siRNA. If we took a close look at the bands in western blotting, we did see a little decrease of p-AKT after Prox1 was knocked by siRNA, because the increase of Prox1 was not higher enough. In our Lab small lung cancer cells have been detected Prox1 200 fold higher than control cells. When Prox1 was knocked down by shRNA- lentivirus, cell proliferation rate was dramatically decreased. In response to intracellular and extracellular stimuli, AKT is highly phosphorylated in order to regulate cell survival and proliferation (25). Notably, Prox1 is an upstream signaling molecule upon AKT signaling pathway, but it is unknown how Prox1 increases phosphor-AKT.

In conclusion, our results suggest that Prox1 increases transfected Prox1-CHO cell proliferation via activation of the AKT signaling pathway. This finding is very helpful to understand why Prox1 promotes tumor progression and metastasis, thereby paving the way for future drug development.
